# The neutrophil-to-lymphocyte ratio independently predicts all-cause mortality in non-dialysis chronic kidney disease patients with preserved red cell distribution width: A retrospective cohort study

**DOI:** 10.1371/journal.pone.0351699

**Published:** 2026-06-22

**Authors:** Yunkyeong Hwang, Janghyun Jo, Suyeon Han, Hwajin Park, Yu Ah Hong, Yoon-Kyung Chang, Dae Eun Choi

**Affiliations:** 1 Department of Internal Medicine, College of Medicine, The Catholic University of Korea, Seoul, Republic of Korea; 2 Department of Nephrology, Daejeon St. Mary’s Hospital, Daejeon, Republic of Korea; 3 Department of Medical Science, Chungnam National University, Daejeon, Republic of Korea; 4 Department of Nephrology, Chungnam National University Hospital, Daejeon, Republic of Korea; University of Diyala College of Medicine, IRAQ

## Abstract

**Background:**

The neutrophil-to-lymphocyte ratio (NLR) and platelet-to-lymphocyte ratio (PLR), inflammatory indices derived from routine complete blood counts, and red cell distribution width (RDW) have been proposed as prognostic markers in chronic kidney disease (CKD). However, whether the inflammatory ratios retain independent prognostic value once erythrocyte homeostasis is considered, and whether their performance varies across the spectrum of erythrocyte heterogeneity captured by RDW, remains uncertain.

**Methods:**

This retrospective cohort study included 2,654 adults with non-dialysis CKD followed at a single tertiary center between 2015 and 2022. Optimal biomarker cut-off values were determined by receiver operating characteristic analysis based on 3-year all-cause mortality. Associations of RDW, NLR, and PLR with dialysis-free survival and overall survival were assessed using Kaplan–Meier analysis and multivariable Cox proportional hazards models adjusted for age, sex, kidney function, comorbidities, and nutritional and metabolic parameters. RDW-stratified analyses and formal interaction testing were performed to to determine whether NLR retains independent prognostic value within RDW-defined subgroups. Parallel analyses restricted to patients with eGFR < 60 mL/min/1.73 m^2^ were performed as a sensitivity analysis and reported as supplementary material.

**Results:**

During a median follow-up of 2,413 days, 451 patients (17.0%) initiated dialysis and 239 (9.0%) died. In multivariable Cox analyses of the whole cohort, NLR did not retain an independent association with either dialysis initiation (HR 1.03, 95% CI 0.84–1.26; p = 0.771) or all-cause mortality (HR 1.23, 95% CI 0.92–1.64; p = 0.162). However, a statistically significant RDW × NLR interaction was observed for overall survival (p for interaction = 0.006): NLR was independently associated with mortality in the low RDW subgroup (HR 2.07, 95% CI 1.25–3.45; p = 0.006) but not in the high RDW subgroup (HR 0.88, 95% CI 0.62–1.26; p = 0.494). PLR did not retain independent prognostic value in any analysis, whereas RDW remained the only marker independently associated with all-cause mortality in the overall multivariable model (HR 1.72, 95% CI 1.31–2.26; p < 0.001). Fine-Gray competing-risks models yielded virtually identical estimates, and findings were consistent in the eGFR < 60 mL/min/1.73 m^2^ subgroup.

**Conclusions:**

In patients with non-dialysis CKD, NLR independently predicts all-cause mortality specifically in patients with preserved RDW, but loses incremental prognostic information once RDW is already elevated. These findings argue against the uncritical use of NLR as a universal prognostic marker in CKD and support an RDW-stratified, outcome-specific framework for applying inflammatory ratios in routine risk stratification.

## Introduction

Chronic kidney disease (CKD) is a growing global public health burden, with a steadily increasing prevalence and a progressive rise in the risks of dialysis initiation and mortality as kidney function declines [1]. The clinical course of CKD is not determined solely by reductions in glomerular filtration rate but also reflects the cumulative effects of chronic inflammation, malnutrition, disordered erythropoiesis, and cardiovascular complications [2,3]. These interrelated processes contribute to both renal disease progression and adverse long-term outcomes.

Among these mechanisms, chronic inflammation has been recognized as a key driver of CKD progression and its complications. Inflammatory activation promotes renal fibrosis, accelerates loss of kidney function, and increases the risk of cardiovascular events, which remain the leading cause of death in patients with CKD [2]. Accordingly, several blood-based inflammatory markers have been proposed as practical tools for risk stratification in this population.

The neutrophil-to-lymphocyte ratio (NLR) and platelet-to-lymphocyte ratio (PLR) are simple, inexpensive indices derived from routine complete blood counts that reflect systemic inflammatory and immune status. NLR integrates neutrophilia, representing innate immune activation, and lymphopenia, reflecting adaptive immune suppression, and has been associated with poor outcomes in various clinical conditions, including cardiovascular disease, malignancy, and CKD [4,5]. PLR has also been suggested as a prognostic marker in several clinical conditions; however, the evidence regarding its predictive value in CKD remains inconsistent, with substantial heterogeneity across studies [6,7].

Red blood cell distribution width (RDW), a measure of variability in erythrocyte size, has traditionally been used in the differential diagnosis of anemia. More recently, elevated RDW has emerged as a robust prognostic marker both in the general population and in patients with chronic diseases. An elevated RDW is thought to reflect the cumulative effects of chronic inflammation, oxidative stress, nutritional deficiency, and impaired erythropoiesis, rather than transient inflammatory activity alone [8–10]. In CKD, where anemia, inflammation, and metabolic disturbances frequently coexist, RDW may therefore capture a broader dimension of physiological vulnerability than inflammatory ratios alone [11].

Despite growing interest in these hematologic indices, studies directly comparing NLR and PLR with RDW within the same CKD population are scarce. Most prior studies have examined these markers in isolation or focused on a single clinical endpoint, and have not consistently applied a uniform multivariable framework with adjustment for the full set of nutritional, anemia, and mineral-metabolism covariates that drive both inflammatory ratios and CKD outcomes [12,13].

In particular, whether the prognostic performance of NLR is uniform across CKD patients or modified by the underlying state of erythrocyte homeostasis—as captured by RDW—has not been systematically examined. Such effect modification would have direct implications for how NLR should be applied in routine risk stratification.

Therefore, this study aimed to evaluate the prognostic performance of NLR for dialysis initiation and all-cause mortality in patients with non-dialysis CKD, in direct head-to-head comparison with PLR and RDW, and to test whether the prognostic value of NLR is modified by RDW strata. We further applied multiple imputation to address missing covariate data, performed Fine-Gray competing-risks sensitivity analyses to ensure robustness, and conducted parallel analyses in the advanced-CKD subgroup with eGFR < 60 mL/min/1.73 m^2^ as a supplementary sensitivity analysis.

## Materials and methods

### Study design and population

This retrospective observational study was conducted at a single center. Adult patients (aged ≥18 years) with chronic kidney disease (CKD) who underwent outpatient evaluation between January 1, 2015, and December 31, 2022, were screened. CKD was defined according to standard clinical criteria based on estimated glomerular filtration rate (eGFR) and/or evidence of kidney damage, such as proteinuria. Patients receiving maintenance dialysis at baseline were excluded.

**Fig 1:**
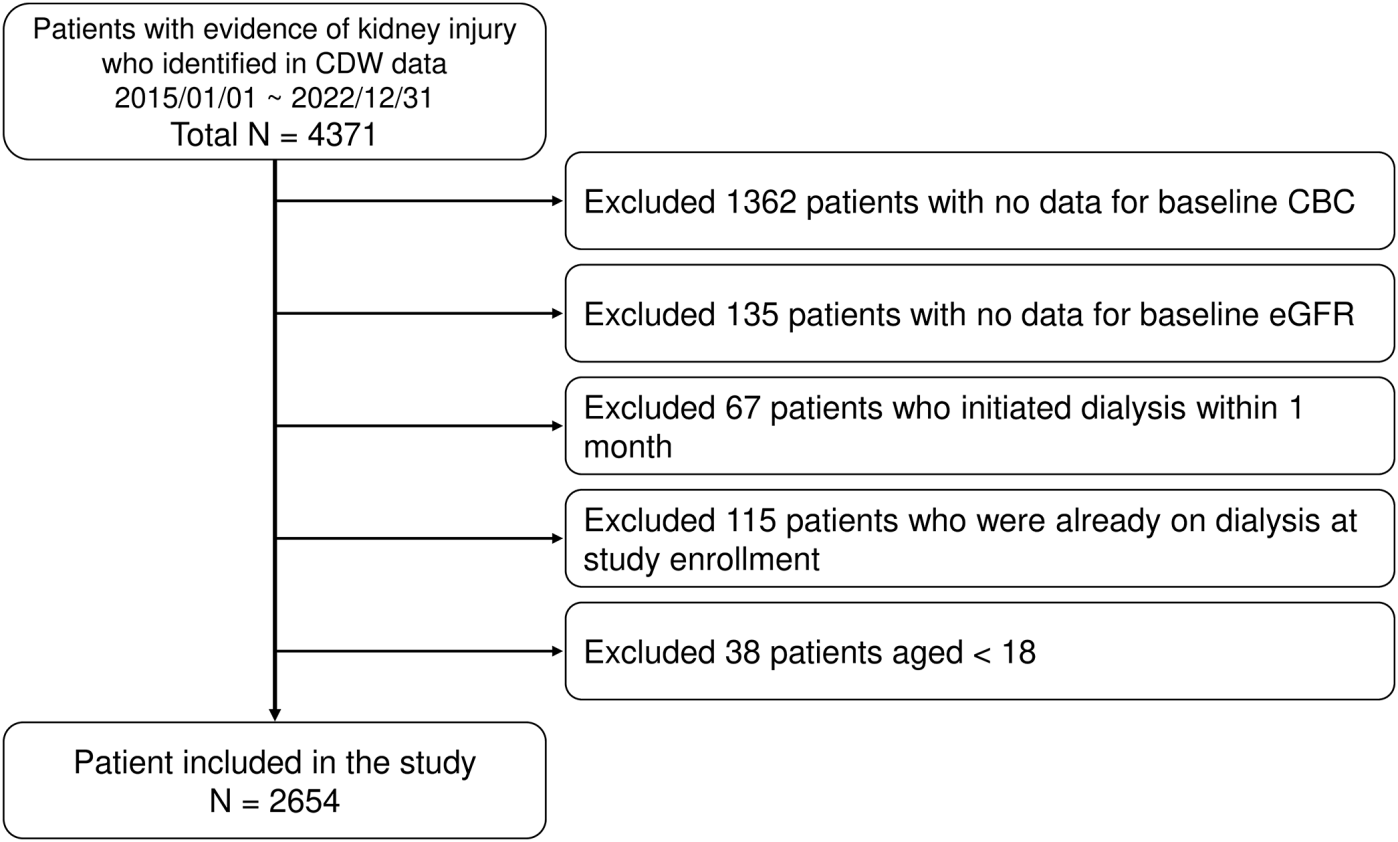
Flowchart of patient inclusion. Flow diagram illustrating patient screening and the selection process for the final study cohort, including the number of patients excluded at each step.

Baseline laboratory measurements were defined as the first available results obtained at study entry. Of the 4,371 patients initially screened, 1,717 were excluded for the following reasons: 1,362 with incomplete baseline complete blood count data, 135 with no baseline estimated glomerular filtration rate, 67 who initiated dialysis within one month of baseline assessment, 115 who were already on dialysis at study entry, and 38 who were younger than 18 years of age. The final analytic cohort comprised 2,654 adult patients with available complete blood count data and follow-up information. The patient selection process, including the number of patients excluded at each step, is summarized in Fig 1. A pre-specified subgroup of patients with eGFR < 60 mL/min/1.73 m^2^ (n = 1,709 was defined for a parallel sensitivity analysis.

### Data collection and variable definitions

Clinical and laboratory data were obtained from the hospital Clinical Data Warehouse (CDW), which was accessed on 28/07/2025 for research purposes. Collected variables included age, sex, comorbidities (diabetes mellitus, hypertension, and cancer), proteinuria, complete blood count parameters, serum albumin, blood urea nitrogen, creatinine, calcium, and phosphorus. Estimated glomerular filtration rate was calculated using the CKD-EPI 2009 equation [14]. Proteinuria was assessed as a binary variable (positive or negative) based on qualitative urinalysis.

The neutrophil-to-lymphocyte ratio (NLR) was calculated as the ratio of absolute neutrophil count to absolute lymphocyte count [15], and the platelet-to-lymphocyte ratio (PLR) as the ratio of platelet count to lymphocyte count [16]. Red blood cell distribution width (RDW) was expressed as RDW–coefficient of variation (RDW-CV). Anemia was defined according to World Health Organization criteria [17].

### Outcomes

The primary outcomes were dialysis-free survival and overall survival. Dialysis-free survival was defined as the time from baseline assessment to dialysis initiation, including the start of maintenance hemodialysis or peritoneal dialysis. Overall survival was defined as the time from baseline assessment to all-cause death. Patients without events were censored at the date of last follow-up.

### Statistical analysis

Continuous variables are presented as median (interquartile range) and categorical variables as number (percentage). Between-group comparisons were performed using the Mann–Whitney U test for continuous variables and the chi-square test for categorical variables, as appropriate.

NLR, PLR, and RDW were treated as continuous variables in descriptive analyses. For survival analyses, each marker was dichotomized into high and low groups using optimal cut-off values derived from receiver operating characteristic (ROC) curve analysis, with the Youden index, applied separately to the 3-year horizons for both dialysis-free and overall survival. Optimal cut-off values were determined by ROC curve analysis with the Youden index (sensitivity + specificity − 1), based on 3-year all-cause mortality as the endpoint (S1 Fig and S1 Table). The cut-off values, applied to both dialysis-free and overall survival analyses, were NLR ≥ 2.14, PLR ≥ 143.14, and RDW ≥ 13.65%. Dichotomization was chosen over continuous modeling to facilitate direct clinical interpretation and risk-group identification.

Dialysis-free survival and overall survival were estimated using the Kaplan–Meier method and compared using the log-rank test. Associations between hematologic markers and clinical outcomes were evaluated using Cox proportional hazards regression models. Multivariable models were adjusted for age, sex, serum albumin, eGFR, diabetes mellitus, hypertension, proteinuria, anemia, calcium, and phosphorus. To minimize potential bias from complete-case analysis, missing covariate values were handled by multiple imputation by chained equations (MICE) with predictive mean matching, generating 20 imputed datasets (m = 20); estimates and 95% confidence intervals were pooled using Rubin’s rules. Interaction between RDW and NLR (and RDW and PLR) was assessed by including a product interaction term in the Cox models. To address the potential dependent-censoring effect of dialysis initiation on mortality, and vice versa, Fine-Gray sub-distribution hazard models were performed as sensitivity analyses for both outcomes in complete-case and MICE-pooled formats. For the low RDW subgroup mortality analysis, where the events-per-variable (EPV) ratio of the full model was limited (EPV = 7.8), a sensitivity analysis using a reduced 4-covariate model (age, sex, albumin, eGFR) was additionally performed. To assess whether the principal findings were preserved in patients with reduced kidney function, all key Kaplan–Meier analyses, multivariable Cox models, and RDW-stratified analyses were repeated in the subgroup with eGFR < 60 mL/min/1.73 m^2^ and are reported as supplementary material.

As this was a retrospective cohort study using all eligible patients identified over the 7-year study period, no formal a priori sample size calculation was performed. The final cohort of 2,654 patients yielded 451 dialysis events and 239 deaths, corresponding to events-per-variable (EPV) ratios of 41.0 and 21.7, respectively, in the primary multivariable models. In the RDW-stratified mortality analysis, the low RDW subgroup contributed only 86 death events (EPV = 7.8), which was addressed by the reduced-model sensitivity analysis described above.

All statistical analyses were performed using R software (version 4.4.2; R Foundation for Statistical Computing, Vienna, Austria). A two-sided p-value <0.05 was considered statistically significant.

### Ethics statement

This study was approved by the Institutional Review Board of Daejeon St. Mary’s Hospital (IRB No. DC25WASB0025). The requirement for informed consent was waived due to the retrospective study design and the use of de-identified data.

## Results

### Patient characteristics

A total of 2,654 patients were included in the study and were classified into low RDW (n = 1,500) and high RDW (n = 1,154) groups according to the predefined RDW cut-off value (S1 Fig). The median age of the overall cohort was 66.0 years (interquartile range [IQR], 53.0–76.0). Baseline characteristics stratified by RDW are summarized in Table 1. Patients in the high-RDW group were significantly older, had higher prevalence of diabetes mellitus and anemia, lower hemoglobin, serum albumin, and eGFR, and higher BUN and creatinine compared with the low-RDW group. The incidence of dialysis initiation (20.9% vs. 14.0%) and mortality (13.8% vs. 5.3%) were both significantly higher in the high-RDW group.

**Table 1:** Baseline characteristics of study population stratified by RDW.

Variable	Total (N = 2654)	Low RDW (N = 1500)	High RDW (N = 1154)	p-value
Age (yr)	66.00 [53.00–76.00]	62.00 [49.00–73.00]	70.00 [60.00–78.00]	<0.001
Male, n (%)	1068 (40.2%)	620 (41.3%)	448 (38.8%)	0.205
DM, n (%)	1313 (49.5%)	694 (46.3%)	619 (53.6%)	<0.001
HTN, n (%)	1166 (43.9%)	663 (44.2%)	503 (43.6%)	0.783
Cancer, n (%)	93 (3.5%)	51 (3.4%)	42 (3.6%)	0. 821
Dialysis, n (%)	451 (17.0%)	210 (14.0%)	241 (20.9%)	<0.001
Time to dialysis (day)	820.00 [384.50–1528.00]	1054.00 [590.50–2004.25]	601.00 [276.00–1256.00]	<0.001
Death, n (%)	239 (9.0%)	80 (5.3%)	159 (13.8%)	<0.001
Time to death (day)	997.00 [529.00–1874.00]	1234.50 [706.50–2347.00]	901.00 [455.00–1717.00]	<0.001
Follow-up duration (day)	2413.00 [1730.00–3223.25]	2467.00 [1743.75–3347.75]	2340.50 [1699.50–3033.50]	<0.001
WBC (10³/mm³)	6.70 [5.50–8.20]	6.60 [5.50–7.93]	6.90 [5.50–8.60]	0.007
Hb (g/dL)	12.40 [10.80–14.10]	13.10 [11.40–14.60]	11.55 [10.10–13.20]	<0.001
Platelet (10³/mm³)	219.50 [180.00–265.00]	222.00 [184.00–263.00]	215.00 [173.00–268.75]	0.053
Neutrophil (10³/mm³)	3.95 [3.04–5.19]	3.79 [3.00–4.91]	4.15 [3.15–5.46]	<0.001
Lymphocyte (10³/mm³)	1.82 [1.40–2.29]	1.88 [1.52–2.36]	1.70 [1.28–2.21]	<0.001
BUN (mg/dL)	21.80 [15.70–32.50]	19.30 [14.60–28.02]	25.80 [18.00–38.00]	<0.001
Creatinine (mg/dL)	1.39 [1.03–2.10]	1.26 [0.93–1.86]	1.60 [1.20–2.55]	<0.001
Albumin (g/dL)	4.40 [4.10–4.70]	4.50 [4.20–4.70]	4.30 [3.90–4.60]	<0.001
Ca (mg/dL)	9.30 [8.90–9.60]	9.40 [9.10–9.70]	9.20 [8.80–9.60]	<0.001
P (mg/dL)	3.60 [3.10–4.00]	3.50 [3.10–4.00]	3.60 [3.20–4.10]	0.005
eGFR CKD-EPI 2009 (mL/min/1.73m²)	46.19 [26.65–74.48]	54.12 [31.67–83.91]	38.81 [21.07–58.80]	<0.001
Proteinuria, positive, n (%)†	1353 (51.0%)	773 (51.5%)	580 (50.3%)	0.556
Anemia, n (%)	1343 (50.6)	585 (39)	758 (65.7)	<0.001
RDW	13.40 [12.90–14.10]	13.00 [12.70–13.30]	14.30 [13.80–15.10]	<0.001
NLR	2.14 [1.54–3.07]	1.99 [1.47–2.80]	2.43 [1.67–3.45]	<0.001
PLR	119.15 [91.43–161.41]	115.79 [90.77–150.65]	125.67 [92.09–175.30]	<0.001

Values are presented as median (interquartile range) or number (%).

p-values were calculated using the Mann–Whitney U test or chi-square test, as appropriate.

Time-to-event variables were calculated from the baseline assessment and are presented for descriptive purposes only.

†Proteinuria was assessed as a binary variable (positive or negative) based on qualitative urinalysis.

### ROC-based cut-offs and unadjusted survival analyses

**Fig 2:**
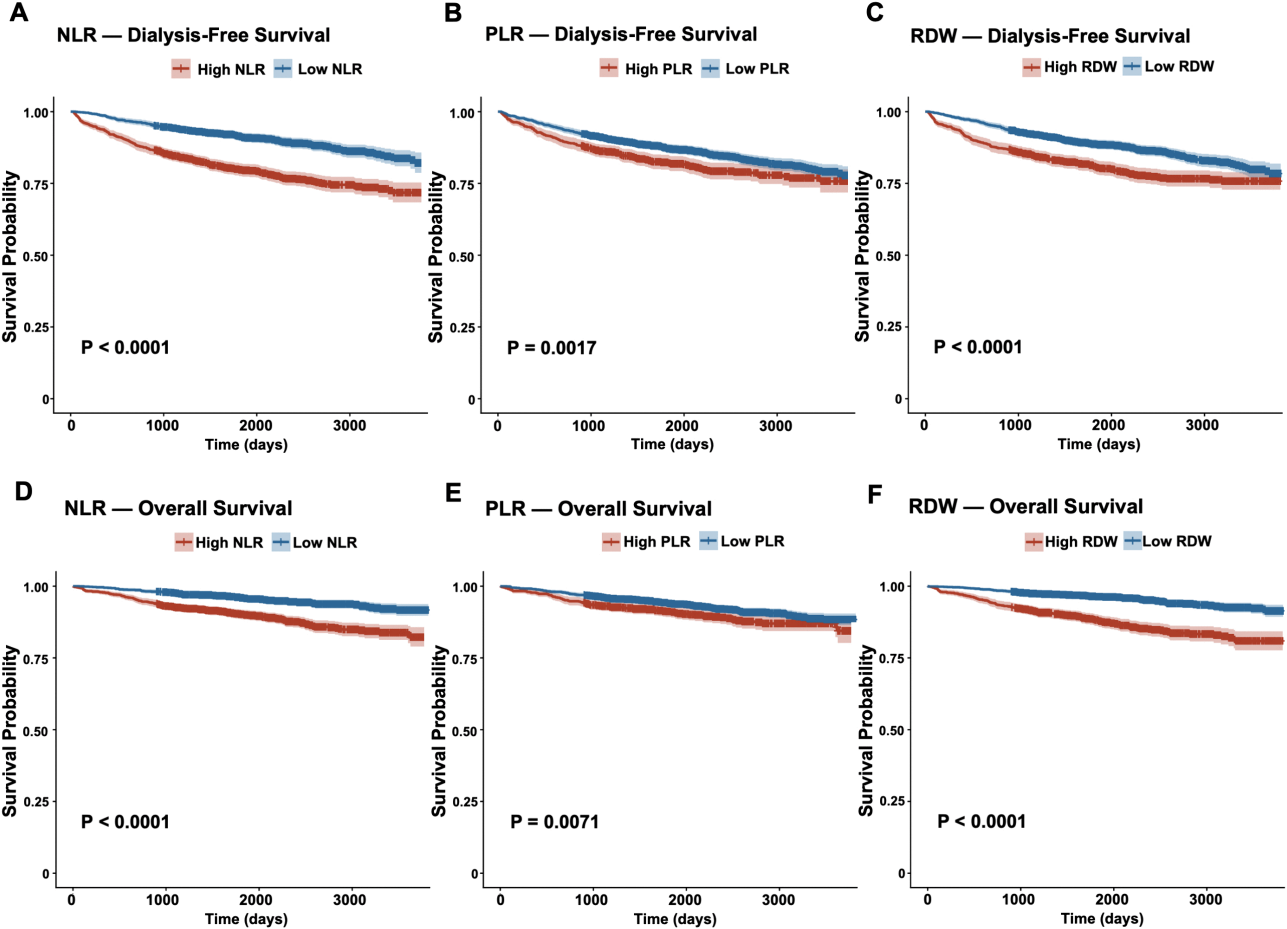
Kaplan–Meier curves for dialysis-free and overall survival stratified by NLR, PLR, and RDW in the overall cohort. Kaplan–Meier curves illustrate dialysis-free survival (top panels, A-C) and overall survival (bottom panels, D-F) according to high and low groups of neutrophil-to-lymphocyte ratio (NLR), platelet-to-lymphocyte ratio (PLR), and red blood cell distribution width (RDW). Biomarker groups were dichotomized using cut-off values derived from receiver operating characteristic (ROC) curve analysis based on the Youden index (NLR ≥ 2.14, PLR ≥ 143.14, RDW ≥ 13.65%). Differences between survival curves were assessed using the log-rank test.

Receiver operating characteristic curve analyses identified the following optimal Youden-index cut-offs based on 3-year all-cause mortality: NLR ≥ 2.14 (AUC = 0.688; sensitivity 0.761, specificity 0.521), PLR ≥ 143.14 (AUC = 0.556; sensitivity 0.477, specificity 0.685), and RDW ≥ 13.65% (AUC = 0.705; sensitivity 0.706, specificity 0.619) (S1 Fig and S1 Table). These cut-off values were applied to both dialysis-free and overall survival analyses. In Kaplan–Meier analyses on the overall cohort, patients in the high-marker groups for NLR, PLR, and RDW showed significantly lower dialysis-free survival and overall survival compared with low-marker counterparts (all log-rank p < 0.01; Fig 2).

In a parallel analysis restricted to patients with eGFR < 60 mL/min/1.73 m^2^ (n = 1,709), NLR retained statistically significant separation for both outcomes (dialysis p < 0.001; mortality p < 0.001). In contrast, PLR no longer significantly stratified either outcome in this subgroup (dialysis p = 0.17; mortality p = 0.21), and RDW retained strong separation for mortality (p < 0.001) but only marginal separation for dialysis-free survival (p = 0.046) (S2 Fig). The qualitative pattern in the advanced-CKD subgroup was therefore consistent with that observed in the whole cohort.

**Fig 3:**
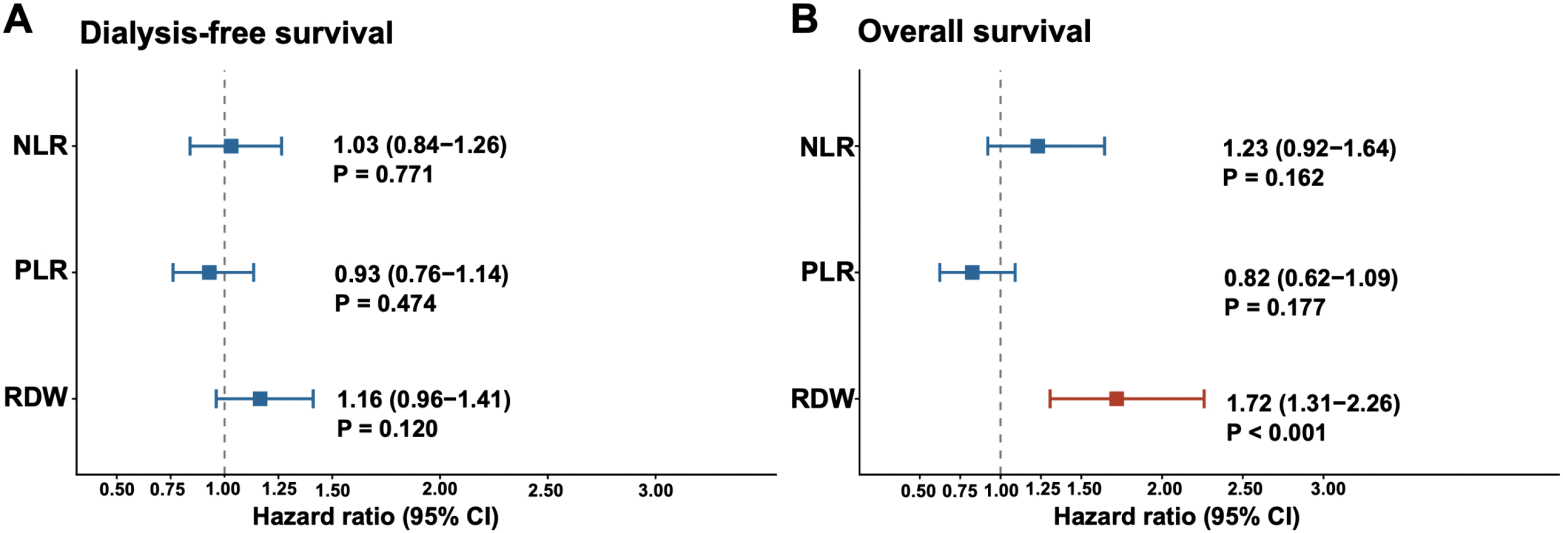
Forest plots showing hazard ratios for dialysis-free and overall survival according to NLR, PLR, and RDW levels in the overall cohort. Forest plots display adjusted hazard ratios (HRs) and 95% confidence intervals (CIs) for high vs low groups of NLR, PLR, and RDW for (A) dialysis-free survival and (B) overall survival. Hazard ratios were estimated using multivariable Cox proportional hazards models adjusted for age, sex, serum albumin, eGFR, diabetes mellitus, hypertension, proteinuria, anemia, calcium, and phosphorus. Missing covariate values were handled by multiple imputation by chained equations (MICE; m = 20, predictive mean matching); estimates and 95% confidence intervals were pooled using Rubin’s rules. Red indicates p < 0.05; blue indicates p ≥ 0.05.

After multivariable adjustment for age, sex, serum albumin, eGFR, diabetes mellitus, hypertension, proteinuria, anemia, calcium, and phosphorus, with missing values handled by MICE (m = 20), none of the three markers retained an independent association with dialysis initiation (NLR: HR 1.03, 95% CI 0.84–1.26, p = 0.771; PLR: HR 0.93, 95% CI 0.76–1.14, p = 0.474; RDW: HR 1.16, 95% CI 0.96–1.41, p = 0.120). For overall survival, neither NLR nor PLR retained statistical significance (NLR: HR 1.23, 95% CI 0.92–1.64, p = 0.162; PLR: HR 0.82, 95% CI 0.62–1.09, p = 0.177), whereas RDW remained the only marker independently associated with all-cause mortality (HR 1.72, 95% CI 1.31–2.26; p < 0.001) (Fig 3). These findings stood in marked contrast to the univariable analyses (S2 and S3 Tables), in which NLR, PLR, and RDW all showed strong associations with both outcomes, indicating that the apparent univariable signals for NLR and PLR were largely attributable to confounding by kidney function, anemia, and metabolic disturbances rather than reflecting independent prognostic contributions. Parallel multivariable analyses restricted to the eGFR < 60 mL/min/1.73 m^2^ subgroup yielded a consistent pattern: NLR and PLR did not retain statistical significance for either outcome, whereas RDW remained the only independent marker for all-cause mortality (S3 Fig). Fine-Gray sub-distribution hazard models, accounting for the competing risk of the alternate outcome (death for the dialysis analysis; dialysis for the mortality analysis), yielded essentially identical estimates in both complete-case and MICE-pooled formats (S4 Table). For overall survival, the RDW sub-distribution hazard ratio in the MICE-pooled Fine-Gray model was closely consistent with the standard Cox estimate, confirming that the dominant prognostic role of RDW for mortality and the absence of independent associations for NLR and PLR were not artifacts of competing risks.

### RDW-stratified analyses: NLR as an independent predictor of mortality in patients with preserved RDW

**Fig 4:**
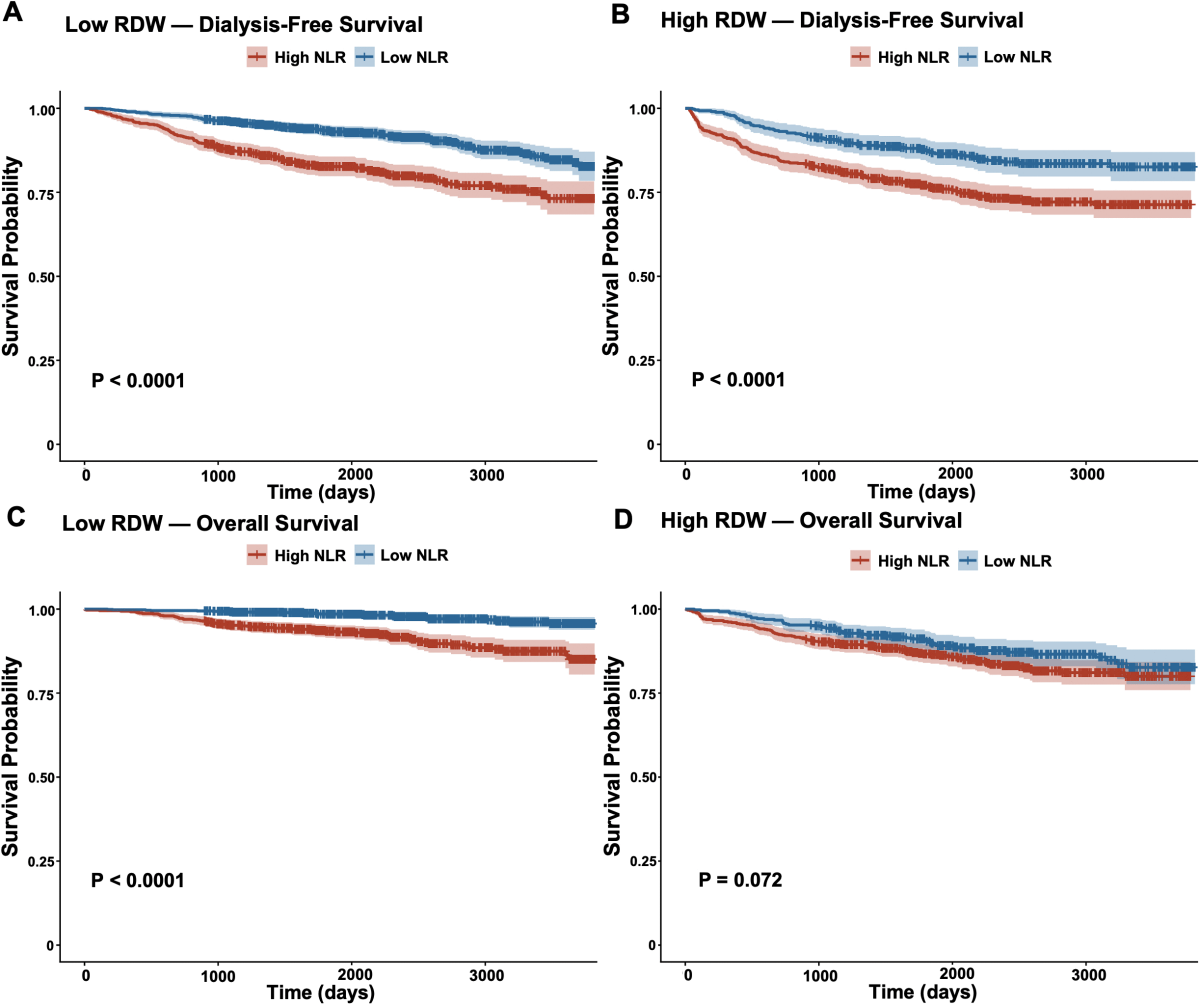
Kaplan–Meier curves for dialysis-free and overall survival stratified by NLR in low-RDW and high-RDW patient groups. Kaplan–Meier curves illustrate dialysis-free survival (top panels, A–B) and overall survival (bottom panels, C–D) according to NLR groups within strata defined by RDW (low RDW [< 13.65%] and high RDW [≥ 13.65]). NLR groups were dichotomized using the pre-specified cut-off of 2.14. Differences between survival curves were assessed using the log-rank test.

To examine whether the prognostic performance of NLR was modified by underlying erythrocyte heterogeneity, RDW-stratified Cox models were fitted. Kaplan–Meier analyses showed distinct temporal patterns of survival curve separation between NLR groups according to RDW strata (Fig 4).

**Fig 5:**
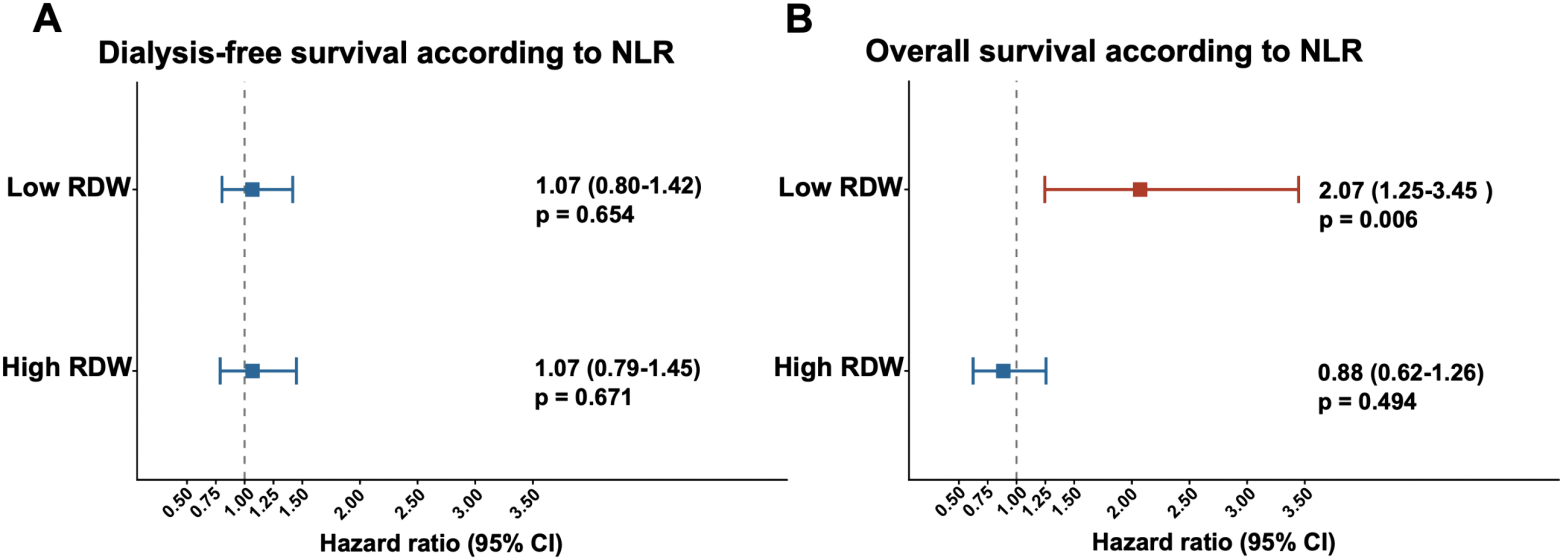
Adjusted hazard ratios for dialysis-free and overall survival according to NLR within low RDW and high RDW subgroups. Forest plots present adjusted hazard ratios (HRs) and 95% confidence intervals (CIs) for high vs. low NLR within low RDW and high RDW subgroups for (A) dialysis-free survival and (B) overall survival. Hazard ratios were estimated using multivariable Cox proportional hazards models adjusted for age, sex, serum albumin, eGFR, diabetes mellitus, hypertension, proteinuria, anemia, calcium, and phosphorus, with MICE-pooled estimates (m = 20, predictive mean matching). Red indicates p < 0.05; blue indicates p ≥ 0.05.

For dialysis-free survival, NLR showed no statistically significant independent association in either the low RDW (HR 1.07, 95% CI 0.80–1.42; p = 0.654) or the high RDW subgroup (HR 1.07, 95% CI 0.79–1.45; p = 0.671), and the RDW × NLR interaction term was not significant. For overall survival, a markedly different pattern was observed, and this represented the principal NLR-related finding of the study. A statistically significant RDW × NLR interaction was detected (p for interaction = 0.006): in the low RDW subgroup, NLR was independently associated with mortality (HR 2.07, 95% CI 1.25–3.45; p = 0.006), whereas in the high RDW subgroup NLR showed no association (HR 0.88, 95% CI 0.62–1.26; p = 0.494) (Fig 5). Given the limited events-per-variable ratio in the low RDW subgroup for mortality (EPV = 7.8), a sensitivity analysis using a reduced 4-covariate model (age, sex, albumin, eGFR; EPV = 21.5) was performed. The NLR–mortality association in the low RDW subgroup remained robust, and the RDW × NLR interaction remained statistically significant, supporting the robustness of this finding (S5 Table). The same pattern of RDW-dependent NLR effect modification was reproduced in the eGFR < 60 mL/min/1.73 m^2^ subgroup (S4 and S5 Figs): NLR was independently associated with mortality in the low RDW subgroup (HR 2.01, 95% CI 1.16–3.48; p = 0.015) but not in the high RDW subgroup (HR 0.89, 95% CI 0.62–1.29; p = 0.552), further supporting the generalizability of this finding to advanced CKD.

RDW-stratified analyses of PLR did not show a consistent pattern of effect modification. For dialysis-free survival, PLR was not significantly associated with the outcome in either stratum (low RDW: HR 0.96, 95% CI 0.72–1.29, p = 0.800; high RDW: HR 0.90, 95% CI 0.68–1.20, p = 0.475), and the RDW × PLR interaction was not significant. For overall survival, PLR also showed no statistically significant association with mortality in either RDW stratum (low RDW: HR 0.92, 95% CI 0.57–1.47, p = 0.723; high RDW: HR 0.72, 95% CI 0.51–1.02, p = 0.068), and the RDW × PLR interaction did not reach statistical significance (S6 and S7 Figs). Consistent results were obtained in the eGFR < 60 mL/min/1.73 m² subgroup (S8 and S9 Figs). Taken together, PLR did not retain independent prognostic value for either outcome within either RDW stratum, and is therefore reported as a comparator marker rather than a primary prognostic candidate.

## Discussion

In this study of patients with non-dialysis CKD, we compared three readily available hematologic indices—NLR, PLR, and RDW—for their prognostic performance against two clinical outcomes (dialysis initiation and all-cause mortality) using a uniform multivariable framework with MICE-based handling of missing covariates and Fine-Gray competing-risks sensitivity analyses. The principal findings are: (i) NLR independently predicts all-cause mortality in patients with preserved RDW (HR 2.07; p = 0.006) but not in those with already-elevated RDW (HR 0.88; p = 0.494), confirmed by a statistically significant RDW × NLR interaction (p = 0.006); NLR identifies a subset of patients with preserved erythrocyte homeostasis (low RDW) at substantially elevated mortality risk but provides no incremental information once RDW is already elevated; (ii) PLR did not retain independent prognostic value in any analysis, regardless of RDW stratum; and (iii) RDW emerged as the only marker independently associated with all-cause mortality across the whole cohort. None of the three markers retained statistical significance for dialysis initiation in the multivariable framework. The same patterns were observed in the eGFR < 60 mL/min/1.73 m^2^ subgroup.

The significant RDW × NLR interaction for mortality is the most clinically actionable finding of this study. In the low RDW subgroup—patients without overt evidence of systemic erythrocyte disturbance—elevated NLR identified a subset at substantially elevated mortality risk (HR 2.07, 95% CI 1.25–3.45; p = 0.006), whereas in the high RDW subgroup NLR provided no incremental information (HR 0.88, 95% CI 0.62–1.26; p = 0.494). Mechanistically, this pattern is biologically coherent: in patients who already exhibit multi-system involvement reflected by elevated RDW, mortality risk is dominated by the broader systemic disturbance that RDW captures [10,11,13], leaving little additional information for an inflammatory ratio to contribute. Conversely, in patients with preserved erythrocyte homeostasis, an elevated NLR may signal an emerging inflammatory burden not yet reflected in red-cell parameters and therefore retains independent prognostic relevance [4,15]. A sensitivity analysis using a reduced 4-covariate model confirmed the robustness of this interaction, and the same pattern was reproduced in the eGFR < 60 mL/min/1.73 m² subgroup, mitigating concerns about the limited events-per-variable ratio in that stratum. This conditional behavior provides a concrete clinical framework for applying NLR: rather than treating it as a universal prognostic index, NLR should be interpreted in light of the patient’s RDW status.

The dominant prognostic role of RDW for all-cause mortality is consistent with a growing body of evidence that RDW reflects an integrated composite of chronic inflammation, oxidative stress, impaired erythropoiesis, and nutritional deficits, rather than any single inflammatory or hematologic axis [8–10,13,18,19]. This biological breadth may explain why RDW captures mortality risk that survives adjustment for the major established CKD-related determinants of survival. In contrast, the loss of statistical significance for NLR and PLR in the overall multivariable model suggests that the strong univariable signals for these inflammatory ratios in CKD may largely reflect shared variance with conventional clinical risk factors—declining eGFR, anemia, hypoalbuminemia, and mineral-metabolism derangement—rather than uniform independent biological contribution across the entire CKD population [2,5,20,21]. For dialysis initiation, the absence of independent associations for all three markers after multivariable adjustment is biologically coherent: in non-dialysis CKD, the trajectory toward renal replacement therapy is overwhelmingly determined by baseline eGFR, proteinuria, and underlying comorbidities, which together capture the dominant drivers of progression [3,14].

For PLR, no statistically significant independent association with either outcome was observed in any analysis—whole-cohort multivariable, RDW-stratified, eGFR < 60 mL/min/1.73 m^2^ subgroup, or interaction testing. Although PLR showed a borderline trend toward lower mortality in the high RDW subgroup (HR 0.72, 95% CI 0.51–1.02, p = 0.068), this did not reach statistical significance and was not corroborated by other analyses. Potential explanations for the limited prognostic performance of PLR in CKD include the multifactorial determinants of platelet counts (iron deficiency, nutritional status, medication exposure, bleeding tendency, marrow dysfunction) and possible competing effects of thrombocytosis as a marker of acute illness versus thrombocytopenia in advanced systemic disease [6,7,22,23]. These findings carry several clinical and methodological implications. First, NLR should not be interpreted as a uniform prognostic marker across non-dialysis CKD; rather, its clinical utility is concentrated in patients with preserved erythrocyte homeostasis (RDW < 13.65%), where it identifies a subset at substantially elevated mortality risk that would otherwise be missed. Second, in patients with already-elevated RDW, NLR adds little incremental information, and risk stratification in this group is better anchored to RDW itself. Third, PLR did not contribute independent prognostic information in any analysis and should not be prioritized as a primary prognostic marker in CKD. Finally, our findings caution against uncritical use of NLR and PLR as universal prognostic markers in CKD without considering effect modification by RDW and without adjustment for the full set of clinical covariates.

### Strengths and limitations

This study has several strengths. The simultaneous head-to-head comparison of NLR, PLR, and RDW within a single non-dialysis CKD cohort allows direct evaluation of their relative prognostic performance, moving beyond the single-marker, single-endpoint paradigm of prior literature. The relatively large sample size (n = 2,654), median follow-up of approximately 6.6 years, and adequate events-per-variable ratios for the primary multivariable models support statistical robustness. The use of MICE-based multiple imputation addresses potential bias from complete-case analysis, and Fine-Gray competing-risks sensitivity analyses confirm that the principal findings are not artifacts of dependent censoring between dialysis initiation and death. Formal interaction testing combined with stratified subgroup analyses provides a structured framework for evaluating effect modification, and parallel analyses restricted to patients with eGFR < 60 mL/min/1.73 m^2^ confirm that the principal findings are preserved in the advanced-CKD subgroup.

The study also has limitations. Its retrospective, single-center design limits causal inference and generalizability, and referral patterns inherent to a tertiary care setting may have introduced selection bias toward patients with more advanced or complicated diseases. Cut-off values were derived and applied within the same cohort, raising the possibility of overfitting; external validation will be important. Information on erythropoiesis-stimulating agent use, iron supplementation, and other medications that may influence RDW or platelet count were not available and could not be adjusted for. Serum vitamin B12 and folate were not routinely measured and could not be incorporated as nutritional adjustment variables; as a result, nutritional contributors to elevate RDW from B12 or folate deficiency could not be specifically excluded. Only baseline biomarker values were analyzed; longitudinal dynamics of NLR, PLR, and RDW were not considered. Finally, the limited number of mortality events in the low RDW subgroup (EPV = 7.8 in the full model) tempers the precision of the NLR–mortality estimate in that stratum, although the sensitivity analysis with a reduced covariate set supports the qualitative finding.

## Conclusions

In this study of patients with non-dialysis CKD, NLR independently predicted all-cause mortality only in patients with preserved erythrocyte homeostasis (low RDW), whereas it provided no incremental information once RDW was already elevated. PLR did not retain independent prognostic value in any analysis, and RDW emerged as the only marker independently associated with mortality across the whole cohort. None of the three markers retained statistical significance for dialysis initiation, consistent with eGFR and proteinuria dominating dialysis-progression risk in this population. These findings argue against the uncritical use of NLR as a universal prognostic marker in CKD and support an RDW-stratified framework in which NLR is applied selectively to patients with preserved erythrocyte homeostasis.

## Supporting information

S1 Fig ROC curve analysis of NLR, PLR, and RDW for predicting dialysis and mortality. Receiver operating characteristic curves for NLR (A, D), PLR (B, E), and RDW (C, F) for 3-year and 5-year dialysis-free survival (top panels) and 3-year overall survival (bottom panels). Optimal cut-off values were determined using the Youden index. A Area under the curve (AUC), cut-off, sensitivity, and specificity are shown in each panel title.
(TIF)

S1 Table ROC-based cut-off values for NLR, PLR, and RDW. Summary of area under the curve (AUC), optimal cut-off values, sensitivity, and specificity derived from ROC analysis for NLR, PLR, and RDW at the 3-year and 5-year time points for dialysis-free survival and overall survival. Cut-off values based on 3-year death events were selected for the primary analyses.
(DOCX)

S2 Fig Kaplan–Meier curves for dialysis-free and overall survival according to NLR, PLR, and RDW in patients with eGFR < 60 mL/min/1.73 m². Kaplan–Meier curves illustrate dialysis-free survival (top panels, A–C) and overall survival (bottom panels, D–F) according to high vs. low groups of NLR (A, D), PLR (B, E), and RDW (C, F) in the subgroup of patients with eGFR < 60 mL/min/1.73 m^2^ (n = 1,709). Biomarker groups were defined using cut-off values derived from ROC curve analysis (NLR ≥ 2.14, PLR ≥ 143.14, RDW ≥ 13.65). NLR retained significant separation for both dialysis-free survival (p < 0.001) and overall survival (p = 0.00057); PLR no longer significantly stratified either outcome (dialysis p = 0.17; mortality p = 0.21); and RDW retained strong separation for mortality (p < 0.0001) but only marginal separation for dialysis-free survival (p = 0.046). Differences between survival curves were compared using the log-rank test.
(TIF)

S2 Table Univariable Cox regression analysis for dialysis-free survival. Unadjusted hazard ratios (HRs) and 95% confidence intervals (CIs) for all covariates included in the primary multivariable models for dialysis-free survival in the whole cohort (n = 2,654).
(DOCX)

S3 Table Univariable Cox regression analysis for overall survival. Unadjusted hazard ratios (HRs) and 95% confidence intervals (CIs) for all covariates included in the primary multivariable models for overall survival in the whole cohort (n = 2,654).
(DOCX)

S3 Fig Forest plots showing adjusted hazard ratios for dialysis-free and overall survival according to NLR, PLR, and RDW in patients with eGFR < 60 mL/min/1.73 m². Forest plots display adjusted hazard ratios (HRs) and 95% confidence intervals (CIs) for high vs low groups of NLR, PLR, and RDW for (A) dialysis-free survival and (B) overall survival, in the subgroup of patients with eGFR < 60 mL/min/1.73 m^2^. Hazard ratios were estimated using multivariable Cox proportional hazards models adjusted for age, sex, serum albumin, eGFR, diabetes mellitus, hypertension, proteinuria, anemia, calcium, and phosphorus, with MICE-pooled estimates (m = 20). Neither NLR nor PLR retained statistical significance for either outcome, whereas RDW remained independently associated with all-cause mortality (HR 1.65, 95% CI 1.24–2.20; p < 0.001). Red indicates p < 0.05; blue indicates p ≥ 0.05.
(TIF)

S4 Table Comparison of standard Cox and Fine-Gray competing-risks models for the principal multivariable analyses. Standard Cox proportional hazards estimates (MICE pooled) and Fine-Gray sub-distribution hazard estimates (complete-case and MICE pooled) for NLR, PLR, and RDW for both dialysis-free survival and overall survival. The competing event was death for the dialysis analysis and dialysis initiation for the mortality analysis. All models were adjusted for age, sex, albumin, eGFR, DM, HTN, proteinuria, anemia, calcium, and phosphorus. Fine-Gray estimates were virtually identical to standard Cox estimates, confirming that the principal findings—particularly the independent association of RDW with mortality—were not artifacts of dependent censoring between the two outcomes.
(DOCX)

S5 Table Sensitivity analysis: NLR and overall survival within RDW strata – full vs. reduced covariate model. Comparison of multivariable hazard ratios for NLR (high vs. low) on overall survival within low RDW and high RDW subgroups, using the full 11-covariate model (age, sex, albumin, eGFR, DM, HTN, proteinuria, anemia, calcium, phosphorus) and a reduced 4-covariate model (age, sex, albumin, eGFR). The reduced model was fitted to address the limited events-per-variable ratio in the low RDW mortality stratum (EPV = 7.8 with 11 covariates vs. EPV = 21.5 with 4 covariates). Both models used MICE-pooled estimates (m = 20, PMM). The NLR–mortality association in the low RDW subgroup and the RDW × NLR interaction remained statistically significant in both models, supporting robustness.
(DOCX)

S4 Fig Kaplan–Meier curves for dialysis-free and overall survival stratified by NLR within low RDW and high RDW subgroups, in patients with eGFR < 60 mL/min/1.73 m². Kaplan–Meier curves illustrate dialysis-free survival (top panels, A–B) and overall survival (bottom panels, C–D) according to high vs low NLR within low RDW and high RDW subgroups, restricted to patients with eGFR < 60 mL/min/1.73 m^2^ (n = 1,709). The same pattern of RDW-dependent NLR effect modification observed in the whole cohort was reproduced in the advanced-CKD subgroup. Cut-offs: NLR ≥ 2.14, RDW ≥ 13.65. Differences between survival curves were assessed using the log-rank test.
(TIF)

S5 Fig Adjusted hazard ratios for dialysis-free and overall survival according to NLR within low RDW and high RDW subgroups, in patients with eGFR < 60 mL/min/1.73 m². Forest plots present adjusted hazard ratios (HRs) and 95% confidence intervals (CIs) for high vs. low NLR within low RDW and high RDW subgroups for (A) dialysis-free survival and (B) overall survival, restricted to patients with eGFR < 60 mL/min/1.73 m^2^. Hazard ratios were estimated using multivariable Cox proportional hazards models adjusted for age, sex, serum albumin, eGFR, diabetes mellitus, hypertension, proteinuria, anemia, calcium, and phosphorus, with MICE-pooled estimates (m = 20). NLR was independently associated with overall mortality in the low RDW subgroup (HR 2.01, 95% CI 1.16–3.48; p = 0.015) but not in the high RDW subgroup (HR 0.89, 95% CI 0.62–1.29; p = 0.552), reproducing the RDW × NLR effect modification observed in the whole cohort. Red indicates p < 0.05; blue indicates p ≥ 0.05.
(TIF)

S6 Fig Kaplan–Meier curves for dialysis-free and overall survival according to PLR within low RDW and high RDW subgroups. Patients were classified by PLR using the cut-off of 143.14 and stratified by RDW status (low RDW [< 13.65] vs high RDW [≥ 13.65]). Kaplan–Meier curves illustrate dialysis-free survival (top panels, A–B) and overall survival (bottom panels, C–D) by PLR group within each RDW stratum, in the whole cohort. In the low RDW subgroup, PLR did not significantly stratify dialysis-free survival (log-rank p = 0.063) but showed significant separation for overall survival (p = 0.012). In the high RDW subgroup, no significant difference was observed for either dialysis-free survival (p = 0.079) or overall survival (p = 0.68).
(TIF)

S7 Fig Adjusted hazard ratios for dialysis-free and overall survival according to PLR within low RDW and high RDW subgroups. Forest plots show adjusted hazard ratios (HRs) and 95% confidence intervals (CIs) for high vs. low PLR within low RDW and high RDW subgroups for (A) dialysis-free survival and (B) overall survival, in the whole cohort. Hazard ratios were estimated using Cox proportional hazards models adjusted for age, sex, serum albumin, eGFR, diabetes mellitus, hypertension, proteinuria, anemia, calcium, and phosphorus, with MICE-pooled estimates (m = 20). Cut-offs: PLR ≥ 143.14, RDW ≥ 13.65. PLR was not significantly associated with either outcome in either RDW stratum, and the RDW × PLR interaction did not reach statistical significance for either endpoint.
(TIF)

S8 Fig Kaplan–Meier curves for dialysis-free and overall survival according to PLR within low RDW and high RDW subgroups, in patients with eGFR < 60 mL/min/1.73 m². Kaplan–Meier curves illustrate dialysis-free survival (top panels, A–B) and overall survival (bottom panels, C–D) according to high vs. low PLR within low RDW and high RDW subgroups, restricted to patients with eGFR < 60 mL/min/1.73 m^2^. PLR did not significantly stratify either dialysis-free survival or overall survival within either RDW stratum in the advanced-CKD subgroup, consistent with the whole-cohort findings. Cut-offs: PLR ≥ 143.14, RDW ≥ 13.65. Differences between survival curves were assessed using the log-rank test.
(TIF)

S9 Fig Adjusted hazard ratios for dialysis-free and overall survival according to PLR within low RDW and high RDW subgroups, in patients with eGFR < 60 mL/min/1.73 m². Forest plots show adjusted hazard ratios (HRs) and 95% confidence intervals (CIs) for high vs. low PLR within low RDW and high RDW subgroups for (A) dialysis-free survival and (B) overall survival, restricted to patients with eGFR < 60 mL/min/1.73 m^2^. Hazard ratios were estimated using multivariable Cox proportional hazards models adjusted for age, sex, serum albumin, eGFR, diabetes mellitus, hypertension, proteinuria, anemia, calcium, and phosphorus, with MICE-pooled estimates (m = 20). PLR did not retain independent prognostic value for either outcome within either RDW stratum, consistent with the whole-cohort findings.
(TIF)

S1 Checklist STROBE statement checklist. Completed STROBE (Strengthening the Reporting of Observational Studies in Epidemiology) checklist for cohort studies, indicating the location in the manuscript where each item is reported.
(PDF)

S1 Data Raw data. Anonymized patient-level dataset underlying all analyses reported in this study.
(XLSX)
