# Supplementary material for: The neutrophil-to-lymphocyte ratio independently predicts all-cause mortality in non-dialysis chronic kidney disease patients with preserved red cell distribution width: A retrospective cohort study
Source: PLoS One. 2026 Jun 22;21(6):e0351699. doi: 10.1371/journal.pone.0351699 (PMC13286174; doi:10.1371/journal.pone.0351699)
Supplement: S1 Table — Summary of area under the curve (AUC), optimal cut-off values, sensitivity, and specificity derived from ROC analysis for NLR, PLR, and RDW at the 3-year and 5-year time points for dialysis-free survival and overall survival. Cut-off values based on 3-year death events were selected for the primary analyses. (DOCX) [file pone.0351699.s002.docx]

S1 Table. ROC-basedcut-off values for NLR, PLR, and RDW.

| Marker | Endpoint | AUC | Cutoff_Youden | Sensitivity | Specificity |
| --- | --- | --- | --- | --- | --- |
| NLR | 3y Dialysis | 0.6763 | 2.3358 | 0.702 | 0.6126 |
| PLR | 3y Dialysis | 0.5747 | 119.1233 | 0.6157 | 0.527 |
| RDW | 3y Dialysis | 0.6058 | 13.55 | 0.6039 | 0.5918 |
| NLR | 3y Death | 0.6881 | 2.1377 | 0.7615 | 0.5212 |
| PLR | 3y Death | 0.5561 | 143.1374 | 0.4771 | 0.6854 |
| RDW | 3y Death | 0.7046 | 13.65 | 0.7064 | 0.619 |
