# Supplementary material for: The neutrophil-to-lymphocyte ratio independently predicts all-cause mortality in non-dialysis chronic kidney disease patients with preserved red cell distribution width: A retrospective cohort study
Source: PLoS One. 2026 Jun 22;21(6):e0351699. doi: 10.1371/journal.pone.0351699 (PMC13286174; doi:10.1371/journal.pone.0351699)
Supplement: S2 Table — Unadjusted hazard ratios (HRs) and 95% confidence intervals (CIs) for all covariates included in the primary multivariable models for dialysis-free survival in the whole cohort (n = 2,654). (DOCX) [file pone.0351699.s004.docx]

S2 Table. Univariable Cox regression analysis for dialysis-free survival.

| Variable | HR (95% CI) | p_value |
| --- | --- | --- |
| RDW (per 1%) | 1.118 (1.057–1.182) | < 0.001 |
| NLR (per 1 unit) | 1.018 (1.006–1.030) | 0.0040 |
| PLR (per 1 unit) | 1.002 (1.001–1.003) | < 0.001 |
| Age (per 1 yr) | 1.002 (0.996–1.008) | 0.4967 |
| Male sex | 0.777 (0.641–0.942) | 0.0102 |
| eGFR (per 1 mL/min/1.73m²) | 0.955 (0.950–0.959) | < 0.001 |
| Albumin (per 1 g/dL) | 0.422 (0.372–0.478) | < 0.001 |
| Diabetes mellitus | 3.110 (2.525–3.831) | < 0.001 |
| Hypertension | 2.115 (1.750–2.557) | < 0.001 |
| Proteinuria | 3.033 (2.459–3.740) | < 0.001 |
| Anemia | 5.418 (4.244–6.917) | < 0.001 |
| Calcium (per 1 mg/dL) | 0.401 (0.358–0.449) | < 0.001 |
| Phosphorus (per 1 mg/dL) | 1.568 (1.462–1.681) | < 0.001 |
