# Supplementary material for: The neutrophil-to-lymphocyte ratio independently predicts all-cause mortality in non-dialysis chronic kidney disease patients with preserved red cell distribution width: A retrospective cohort study
Source: PLoS One. 2026 Jun 22;21(6):e0351699. doi: 10.1371/journal.pone.0351699 (PMC13286174; doi:10.1371/journal.pone.0351699)
Supplement: S3 Table — Unadjusted hazard ratios (HRs) and 95% confidence intervals (CIs) for all covariates included in the primary multivariable models for overall survival in the whole cohort (n = 2,654). (DOCX) [file pone.0351699.s005.docx]

S3 Table. Univariable Cox regression analysis for overall survival.

| Variable | HR (95% CI) | p_value |
| --- | --- | --- |
| RDW (per 1%) | 1.239 (1.167–1.315) | < 0.001 |
| NLR (per 1 unit) | 1.030 (1.017–1.042) | < 0.001 |
| PLR (per 1 unit) | 1.001 (1.000–1.002) | 0.0035 |
| Age (per 1 yr) | 1.063 (1.051–1.075) | < 0.001 |
| Male sex | 1.042 (0.806–1.347) | 0.7534 |
| eGFR (per 1 mL/min/1.73m²) | 0.976 (0.971–0.981) | < 0.001 |
| Albumin (per 1 g/dL) | 0.368 (0.312–0.434) | < 0.001 |
| Diabetes mellitus | 1.326 (1.027–1.711) | 0.0305 |
| Hypertension | 0.962 (0.745–1.243) | 0.7685 |
| Proteinuria | 0.917 (0.711–1.182) | 0.5033 |
| Anemia | 3.531 (2.608–4.782) | < 0.001 |
| Calcium (per 1 mg/dL) | 0.458 (0.389–0.539) | < 0.001 |
| Phosphorus (per 1 mg/dL) | 1.166 (0.999–1.362) | 0.0511 |
