# Supplementary material for: The neutrophil-to-lymphocyte ratio independently predicts all-cause mortality in non-dialysis chronic kidney disease patients with preserved red cell distribution width: A retrospective cohort study
Source: PLoS One. 2026 Jun 22;21(6):e0351699. doi: 10.1371/journal.pone.0351699 (PMC13286174; doi:10.1371/journal.pone.0351699)
Supplement: S4 Table — Standard Cox proportional hazards estimates (MICE pooled) and Fine-Gray sub-distribution hazard estimates (complete-case and MICE pooled) for NLR, PLR, and RDW for both dialysis-free survival and overall survival. The competing event was death for the dialysis analysis and dialysis initiation for the mortality analysis. All models were adjusted for age, sex, albumin, eGFR, DM, HTN, proteinuria, anemia, calcium, and phosphorus. Fine-Gray estimates were virtually identical to standard Cox estimates, confirming that the principal findings—particularly the independent association of RDW with mortality—were not artifacts of dependent censoring between the two outcomes. (DOCX) [file pone.0351699.s007.docx]

S4 Table. Comparison of standard Cox and Fine-Gray competing-risks models for the principal multivariable analyses

| Analysis | RDW stratum | N events | Covariates | EPV | NLR HR (95% CI) | p | p for interaction |
| --- | --- | --- | --- | --- | --- | --- | --- |
| NLR | Dialysis-free survival | 1.03 (0.84–1.26) | 0.778 | 1.03 (0.81–1.30) | 0.810 | 1.04 (0.83–1.29) | 0.742 |
| PLR | Dialysis-free survival | 0.93 (0.76–1.13) | 0.446 | 0.94 (0.75–1.19) | 0.610 | 0.93 (0.75–1.15) | 0.491 |
| RDW | Dialysis-free survival | 1.16 (0.96–1.41) | 0.123 | 1.16 (0.94–1.44) | 0.160 | 1.15 (0.94–1.40) | 0.171 |
| NLR | Overall survival | 1.23 (0.92–1.65) | 0.160 | 1.36 (0.99–1.86) | 0.059 | 1.23 (0.92–1.65) | 0.167 |
| PLR | Overall survival | 0.82 (0.62–1.09) | 0.174 | 0.91 (0.67–1.24) | 0.550 | 0.82 (0.62–1.09) | 0.179 |
| RDW | Overall survival | 1.72 (1.31–2.27) | **<0.001** | 1.69 (1.27–2.26) | **<0.001** | 1.71 (1.29–2.25) | **<0.001** |
