# Supplementary material for: The neutrophil-to-lymphocyte ratio independently predicts all-cause mortality in non-dialysis chronic kidney disease patients with preserved red cell distribution width: A retrospective cohort study
Source: PLoS One. 2026 Jun 22;21(6):e0351699. doi: 10.1371/journal.pone.0351699 (PMC13286174; doi:10.1371/journal.pone.0351699)
Supplement: S5 Table — Comparison of multivariable hazard ratios for NLR (high vs. low) on overall survival within low RDW and high RDW subgroups, using the full 11-covariate model (age, sex, albumin, eGFR, DM, HTN, proteinuria, anemia, calcium, phosphorus) and a reduced 4-covariate model (age, sex, albumin, eGFR). The reduced model was fitted to address the limited events-per-variable ratio in the low RDW mortality stratum (EPV = 7.8 with 11 covariates vs. EPV = 21.5 with 4 covariates). Both models used MICE-pooled estimates (m = 20, PMM). The NLR–mortality association in the low RDW subgroup and the RDW × NLR interaction remained statistically significant in both models, supporting robustness. (DOCX) [file pone.0351699.s008.docx]

S5 Table. Sensitivity analysis: NLR and overall survival within RDW strata – full vs. reduced covariate model.

| Analysis | RDW stratum | N events | Covariates | EPV | NLR HR (95% CI) | p | p for interaction |
| --- | --- | --- | --- | --- | --- | --- | --- |
| Full model (11 covariates) | Low RDW | 86 | 11 | 7.8 | 2.04 (1.21–3.43) | 0.008 | 0.004 |
| Full model (11 covariates) | High RDW | 153 | 11 | 13.9 | 0.88 (0.62–1.25) | 0.480 |  |
| Reduced model (4 covariates) | Low RDW | 86 | 4 | 21.5 | 2.01 (1.21–3.33) | 0.008 | 0.002 |
| Reduced model (4 covariates) | High RDW | 153 | 4 | 38.2 | 0.91 (0.64–1.29) | 0.579 |  |
