# Supplementary material for: The neutrophil-to-lymphocyte ratio independently predicts all-cause mortality in non-dialysis chronic kidney disease patients with preserved red cell distribution width: A retrospective cohort study
Source: PLoS One. 2026 Jun 22;21(6):e0351699. doi: 10.1371/journal.pone.0351699 (PMC13286174; doi:10.1371/journal.pone.0351699)
Supplement: S1 Checklist — Completed STROBE (Strengthening the Reporting of Observational Studies in Epidemiology) checklist for cohort studies, indicating the location in the manuscript where each item is reported. (PDF) [file pone.0351699.s015.pdf]

STROBE Statement — Checklist of items that should be included in reports of cohort studies

Manuscript ID: PONE-D-26-14613

Title: The neutrophil-to-lymphocyte ratio independently predicts all-cause mortality in non-dialysis chronic kidney disease patients with preserved red cell distribution width: a retrospective cohort study

Item No.	Recommendation	Reported in
**Title and abstract**		
1	**(a)** Indicate the study's design with a commonly used term in the title or the abstract<br>**(b)** Provide in the abstract an informative and balanced summary of what was done and what was found	*Title; Abstract*
**Introduction**		
2	Explain the scientific background and rationale for the investigation being reported	*Introduction, paragraphs 1–4*
3	State specific objectives, including any prespecified hypotheses	*Introduction, final paragraph*
**Methods**		
4	Present key elements of study design early in the paper	*Methods — Study design and population*
5	Describe the setting, locations, and relevant dates, including periods of recruitment, exposure, follow-up, and data collection	*Methods — Study design and population*
6	**(a)** Cohort study — Give the eligibility criteria, and the sources and methods of selection of participants; describe methods of follow-up<br>**(b)** Cohort study — For matched studies, give matching criteria and number of exposed and unexposed (not applicable; unmatched cohort)	*Methods — Study design and population; Fig 1*
7	Clearly define all outcomes, exposures, predictors, potential confounders, and effect modifiers; give diagnostic criteria, if applicable	*Methods — Data collection and variable definitions; Outcomes*
8*	For each variable of interest, give sources of data and details of methods of assessment (measurement); describe comparability of assessment methods if there is more than one group	*Methods — Data collection and variable definitions*
9	Describe any efforts to address potential sources of bias	*Methods — Statistical analysis (MICE; Fine-Gray)*
10	Explain how the study size was arrived at	*Methods — Statistical analysis (final paragraph)*
11	Explain how quantitative variables were handled in the analyses; if applicable, describe which groupings were chosen and why	*Methods — Statistical analysis (ROC-derived dichotomization)*
12	**(a)** Describe all statistical methods, including those used to control for confounding<br>**(b)** Describe any methods used to examine subgroups and interactions	*Methods — Statistical analysis*

Item No.	Recommendation	Reported in
	**(c)** Explain how missing data were addressed<br>**(d)** Cohort study — If applicable, explain how loss to follow-up was addressed<br>**(e)** Describe any sensitivity analyses	
**Results**		
**13***	**(a)** Report numbers of individuals at each stage of study — e.g., numbers potentially eligible, examined for eligibility, confirmed eligible, included in the study, completing follow-up, and analysed<br>**(b)** Give reasons for non-participation at each stage<br>**(c)** Consider use of a flow diagram	*Results — Patient characteristics; Fig 1*
**14***	**(a)** Give characteristics of study participants (e.g., demographic, clinical, social) and information on exposures and potential confounders<br>**(b)** Indicate number of participants with missing data for each variable of interest<br>**(c)** Cohort study — Summarise follow-up time (e.g., average and total amount)	*Results — Patient characteristics; Table 1*
**15***	Cohort study — Report numbers of outcome events or summary measures over time	*Results — Patient characteristics (451 dialysis, 239 deaths)*
**16**	**(a)** Give unadjusted estimates and, if applicable, confounder-adjusted estimates and their precision (e.g., 95% confidence interval); make clear which confounders were adjusted for and why they were included<br>**(b)** Report category boundaries when continuous variables were categorized<br>**(c)** If relevant, consider translating estimates of relative risk into absolute risk for a meaningful time period	*Results — ROC-based cut-offs and unadjusted survival; Fig 2, Fig 3; S2 Table, S3 Table*
**17**	Report other analyses done — e.g., analyses of subgroups and interactions, and sensitivity analyses	*Results — RDW-stratified analyses; S4 Table; S5 Table; S2–S5, S8–S9 Figs*
**Discussion**		
**18**	Summarise key results with reference to study objectives	*Discussion, first paragraph*
**19**	Discuss limitations of the study, taking into account sources of potential bias or imprecision; discuss both direction and magnitude of any potential bias	*Discussion — Strengths and limitations*
**20**	Give a cautious overall interpretation of results considering objectives, limitations, multiplicity of analyses, results from similar studies, and other relevant evidence	*Discussion, paragraphs 2–4*
**21**	Discuss the generalisability (external validity) of the study results	*Discussion — Strengths and limitations*
**Other information**		
**22**	Give the source of funding and the role of the funders for the present study and, if applicable, for the original study on which the present article is based	*Funding*

**Give information separately for exposed and unexposed groups. Note: An Explanation and Elaboration article discusses each checklist item and gives methodological background and published examples of transparent reporting. The STROBE checklist is best used in conjunction with this article (freely available at www.annals.org, www.epidem.com, www.plosmedicine.org). Information on the STROBE Initiative is available at www.strobe-statement.org.*
